# Treatment Strategy According to Findings on Pressure-Flow Study for Women with Decreased Urinary Flow Rate

**DOI:** 10.1155/2009/782985

**Published:** 2009-10-13

**Authors:** Yoshinori Tanaka, Naoya Masumori, Taiji Tsukamoto, Seiji Furuya, Ryoji Furuya, Hiroshi Ogura

**Affiliations:** ^1^Department of Urology, Hokkaido Prefectural Esashi Hospital, Japan; ^2^Department of Urology, Sapporo Medical University School of Medicine, Sapporo 060-8543, Japan; ^3^Department of Urology, Furuya Hospital, Kitami 090-0065, Japan

## Abstract

*Purpose*. In women who reported a weak urinary stream, the efficacy of treatment chosen according to the urodynamic findings on pressure-flow study was prospectively evaluated. *Materials and Methods*. Twelve female patients with maximum flow rates of 10 mL/sec or lower were analyzed in the present study. At baseline, all underwent pressure-flow study to determine the degree of bladder outlet obstruction (BOO) and status of detrusor contractility on Schäfer's diagram. Distigmine bromide, 10 mg/d, was given to the patients with detrusor underactivity (DUA) defined as weak/very weak contractility, whereas urethral dilatation was performed using a metal sound for those with BOO (linear passive urethral resistance relation 2–6). Treatment efficacy was evaluated using the International Prostate Symptom Score (IPSS), uroflowmetry, and measurement of postvoid residual urine volume. Some patients underwent pressure-flow study after treatment. *Results*. Urethral dilatation was performed for six patients with BOO, while distigmine bromide was given to the remaining six showing DUA without BOO. IPSS, QOL index, and the urinary flow rate were significantly improved in both groups after treatment. All four of the patients with BOO and one of the three with DUA but no BOO who underwent pressure-flow study after treatment showed decreased degrees of BOO and increased detrusor contractility, respectively. *Conclusions*. Both BOO and DUA cause a decreased urinary flow rate in women. In the short-term, urethral dilatation and distigmine bromide are efficacious for female patients with BOO and those with DUA, respectively.

## 1. Introduction

Pressure-flow study is the only method to simultaneously evaluate bladder outlet obstruction (BOO) and detrusor contractility [1]. It has been demonstrated that there are two major causes of decreased urinary flow rate in women; BOO and detrusor underactivity (DUA), similar to men [2, 3]. Alpha 1 blockers [4] or urethral dilatation may be efficacious by reducing the degree of BOO in women. On the other hand, bethanechol chloride and distigmine bromide may increase the urinary flow rate through improved detrusor contractility in female patients with DUA. However, few studies have investigated the efficacy of treatment strategies decided according to the cause of the decreased urinary flow rate in women.

In the present study, we performed a pressure-flow study for female patients who reported a weak urinary stream to determine the cause of the decreased urinary flow rate. According to the cause, BOO, or DUA, urethral dilatation or medical therapy with distigmine bromide was chosen, and the treatment efficacy was prospectively evaluated.

## 2. Materials and Methods

Female patients who visited Furuya Hospital because of awareness of a weak urinary stream were investigated in the present study. Women having obvious neurogenic bladder, pelvic organ prolapsed, and a history of surgery of the pelvic organs were excluded from the study. Patients received examination with the International Prostate Symptom Score (IPSS), QOL index, uroflowmetry, and measurement of postvoid residual urine volume (PVR). Twelve patients with maximum flow rates (Qmax) of 10 mL/sec or lower were analyzed in the present study. Pressure-flow study was performed after the risks and benefits of the study were explained and the patients gave oral informed consent.

The method used for the pressure-flow study was previously reported [5]. Urodynamic parameters used in the study were based on the standard terminology of the International Continence Society [6]. The urethral resistance factor [7] and maximum watts factor [8] were automatically calculated using a computer [9]. Since there is no consensus on how to properly determine BOO and detrusor contractility in women, we employed Schäfer's diagram [10], which is basically applicable only to men, for female subjects. A linear passive urethral resistance relation (LinPURR) of grade 2 or more and weak/very weak contractility were defined as BOO and DUA, respectively.

The treatment strategy was chosen according to the urodynamic findings. For patients with BOO, the urethra was dilated using a metal sound up to 28 French for 10–15 minutes under urethral anesthesia with 2% xylocaine jelly. For patients with DUA without BOO, distigmine bromide (10 mg/d) was prescribed.

After two weeks of urethral dilatation and four weeks of distigmine bromide treatment, the short-term efficacy was evaluated using the IPSS, QOL index, uroflowmetry, and measurement of PVR. Four of the six patients treated by urethral dilatation and one of the three women treated with distigmine bromide agreed with examination by pressure-flow study after treatment.

Statistical comparisons of unpaired and paired groups were done using the Mann-Whitney *U* test and Wilcoxon signed-rank test, respectively. Statistical significance was assumed at *P* < .05.

## 3. Results

Of the 12 women for whom pressure-flow study was performed, six had BOO (Table 1). They had normal detrusor contractility except one with weak detrusor contractility. All of the remaining six patients without BOO had weak detrusor contractility according to Schäfer's diagram. The mean age of patients without BOO was significantly higher than that of patients with BOO. The urethral resistance factor in patients with BOO was significantly higher than that of those without BOO by definition. On the other hand, the maximum watts factor was significantly lower in patients without BOO than in those with it. There were no significant differences in the IPSS, QOL index, urinary flow rate, and PVR at baseline between patients with and without BOO.

In women with BOO, urethral dilatation significantly improved the IPSS, QOL index, urinary flow rate, and PVR (Table 2). The urethral resistance factor was decreased from 53.2 to 20.1 cm of water, although it did not reach statistical significance. Pressure-flow study after treatment demonstrated that all had a decreased grade of LinPURR (Table 4). In the women without BOO, distigmine bromide significantly improved the IPSS, QOL index, and urinary flow rate (Table 3). The maximum watts factor was slightly increased from 6.1 to 8.9 W/m^2^, but the increase did not reach statistical significance because of small number of patients. Pressure-flow study after treatment demonstrated that one patient had improvement of detrusor contractility from weak to normal (Table 4). No change in the grade of LinPURR was observed in patients taking distigmine bromide nor were any obvious side effects observed during their drug therapy.

## 4. Discussion

There are few reports in which the causes of difficulty on urination of female patients were evaluated by urodynamics including pressure-flow study. Wheeler Jr. et al. [2] reported that the major cause of urinary retention or a large volume of PVR was poor detrusor contractility. On the other hand, Massey and Abrams [3] reported in their retrospective study evaluating urodynamic results of 5948 female patients that difficulty on urination in women was mainly induced by DUA because only 163 patients (2.74%) had BOO. In the present study, half of the female patients with a decreased urinary flow rate had BOO, mostly without DUA, in the pressure-flow study. On the other hand, all patients without BOO exhibited DUA with a lower maximum watts factor than those with BOO. Thus, there were two main causes of the decreased urinary flow rate in female patients: BOO and DUA. Since there were no differences in the IPSS, QOL index, urinary flow rate, and PVR between patients with and without BOO, except age at baseline, it was difficult to determine the existence of BOO only by clinical parameters.

There are several causes of BOO in female patients, including urethral stricture, meatal stenosis, atrophic changes of the vaginal wall, urethral caruncle, extramural compression by pelvic masses, and iatrogenic causes after operation for stress urinary incontinence [3]. Kuo [11] reported that 207 women with BOO were categorized into five groups on the basis of the videourodynamic findings. The study revealed bladder neck obstruction in 18 patients (9%), urethral sphincter obstruction in 56 (27%), pelvic floor muscle obstruction in 106 (51%), pelvic organ prolapse in 13 (6%), and urethral stricture in 14 (7%). It has been reported that women with BOO are treated with urethral dilatation, otis urethrotomy, intermittent self-catheterization, excision of the caruncle, and surgery for extramural obstruction [3]. Diokno et al. [12] and Axelrod and Blaivas [13] reported that female patients with bladder neck obstruction were successfully treated with transurethral neck incision. Botulinum A toxin injection into the urethral sphincter also had potential to decrease urethral resistance and improve voiding [14]. In our study, the causes of BOO were unclear because of lack of videourodynamic evaluation urethral dilatation using a metal sound improved lower urinary tract symptoms (LUTSs) and the urinary flow rate in female patients with BOO. Although it did not reach statistical significance, the urethral resistance factor was reduced. Posttreatment pressure-flow study showed that LinPURR was improved by two to three grades. Thus, urethral dilatation was an effective treatment method for female patients with BOO, although the durability was not evaluated in this study.

Wheeler Jr. et al. [2] reported that weak detrusor contractility was induced by diabetes mellitus, multiple sclerosis, disc hernia, tumors in the central nervous system, total abdominal hysterectomy, and psychosocial problems. The causes of DUA were unclear in our study because women with obvious neurogenic bladder and a history of pelvic surgery were excluded from the study. Since the patients with DUA but no BOO were significantly older than those with BOO, aging of the detrusor muscle might be involved in the development of DUA.

It has been demonstrated that distigmine bromide increases detrusor contractility by inactivating cholinesterase and maintaining cholinergic stimulation. Indeed, in poor male voiders after transurethral resection of the prostate with DUA proven by pressure-flow study, distigmine bromide improved LUTS and the urinary flow rate by increasing detrusor contractility [4]. In the present study, 10 mg of distigmine bromide also improved LUTS and the flow rate in female patients with DUA was proven by pressure-flow study. Although it did not reach statistical significance because of small number of patients, the maximum watts factor was slightly increased after administration of distigmine bromide. Thus, distigmine bromide may be considered for women with DUA with care of development of cardiovascular effects such as flushing, palpitation, and blood pressure elevation and gastrointestinal effects such as nausea, vomiting, diarrhea, and clumps, although the available studies do not strongly support the use of parasympathomimetics for treating DUA because of inconsistent results [15].

Several criteria for female BOO have been proposed [16–19]. Blaivas and Groutz [19] constructed a BOO nomogram for women with LUTS in 2000. Since our study was performed before publication of the nomogram, we could not use it. If the Blaivas and Groutz nomogram had been applied to our patients, five of the six patients with BOO on Schäfer's diagram would have been categorized into moderate or severe obstruction on the nomogram and all six patients without BOO as having no or mild obstruction. Thus, since most of the patients treated with urethral dilatation had moderate or severe obstruction on the Blaivas and Groutz nomogram, selection of the treatment strategy in this study was thought to be suitable.

## 5. Conclusions

The two major causes of decreased urinary flow rate in women were BOO and DUA. In the short-term, urethral dilatation and distigmine bromide improved BOO and detrusor contractility in patients with BOO and DUA, respectively.

## Figures and Tables

**Table 1:** Baseline parameters in patients with and without bladder outlet obstruction.

Parameters	Without BOO^(1)^	With BOO	*P*-value^(2)^
	(*n* = 6)	(*n* = 6)	
Age	69.5 (8.0)^(3)^	46.0 (12.4)	.025
IPSS	17.8 (8.3)	20.0 (7.6)	.471
QOL index	5.3 (0.5)	5.7 (0.5)	.337
Uroflowmetry			
* *Voided volume (mL)	223.7 (54.6)	176.7 (95.0)	.423
* *Maximum flow rate (mL/sec)	8.1 (2.3)	6.8 (3.2)	.379
* *Average flow rate (mL/sec)	3.7 (1.0)	3.1 (1.7)	.471
* *Postvoid residual urine volume (mL)	71.3 (69.4)	236.3 (170.5)	.078

Pressure flow studies			
* *Maximum flow rate (mL/sec)	8.3 (2.3)	5.8 (1.5)	.093
* *Pressure at maximum flow (cm water)	23.2 (11.6)	88.0 (20.2)	.004
* *Urethral resistance factor (cm water)	15.4 (8.0)	52.9 (11.3)	.004
* *Maximum watts factor (W/m^2^)	5.2 (2.1)	12.4 (2.9)	.004

^(1)^BOO, bladder outlet obstruction ^(2)^Mann-Whitney *U* test ^(3)^Mean (standard deviation).

**Table 2:** Change in parameters after urethral dilatation in patients with bladder outlet obstruction.

Parameters	Pretreatment	Posttreatment	*P*-value^(1)^
Clinical parameters (*n* = 6)			
* *IPSS	20.0 (7.6)^(2)^	4.8 (0.8)	.028
* *QOL index	5.7 (0.5)	2.2 (1.0)	.028
* *Uroflowmetry			
* *Voided volume (mL)	176.7 (95.0)	222.0 (54.1)	.028
* *Maximum flow rate (mL/sec)	6.8 (3.2)	13.9 (4.9)	.028
* *Average flow rate (mL/sec)	3.1 (1.7)	7.9 (3.1)	.028
* *Postvoid residual urine volume (mL)	236.3 (170.5)	80.8 (102.0)	.028

Pressure flow studies (*n* = 4)			
* *Maximum flow rate (mL/sec)	5.2 (1.5)	14.2 (3.4)	.068
* *Pressure at maximum flow (cm water)	80.5 (18.0)	49.8 (18.9)	.068
* *Urethral resistance factor (cm water)	53.2 (14.2)	20.1 (4.9)	.068
* *Maximum watts factor (W/m^2^)	11.4 (2.8)	11.2 (3.7)	<.999

^(1)^Wilcoxon signed-rank test ^(2)^Mean (standard deviation).

**Table 3:** Change in parameters after distigmine bromide treatment in patients without bladder outlet obstruction.

Parameters	Pretreatment	Posttreatment	*P*-value^(1)^
Clinical parameters (*n* = 6)			
* *IPSS	17.8 (8.3)^(2)^	5.2 (4.8)	.028
* *QOL index	5.3 (0.5)	1.3 (1.0)	.028
* *Uroflowmetry			
* *Voided volume (mL)	223.7 (54.6)	245.7 (124.6)	.753
* *Maximum flow rate (mL/sec)	8.1 (2.3)	13.1 (4.3)	.046
* *Average flow rate (mL/sec)	3.7 (1.0)	6.8 (1.8)	.028
* *Postvoid residual urine volume (mL)	71.3 (69.4)	45.0 (61.0)	.249

Pressure flow studies (*n* = 3)			
* *Maximum flow rate (mL/sec)	8.3 (2.9)	13.3 (0.6)	.109
* *Pressure at maximum flow (cm water)	31.0 (5.6)	33.0 (8.7)	<.999
* *Urethral resistance factor (cm water)	20.2 (1.3)	15.8 (2.1)	.109
* *Maximum watts factor (W/m^2^)	6.1 (2.5)	8.9 (0.2)	.109

^(1)^Wilcoxon signed-rank test ^(2)^Mean (standard deviation).

**Table:** (a) Urethral dilatation in patients with BOO^(1)^ (*n* = 4).

	LinPURR^(2)^ (Detrusor contractility)	
	Pre-treatment	Post-treatment
Patient 1	degree 4 (Normal)	degree 2 (Strong)
Patient 2	degree 4 (Normal)	degree 2 (Normal)
Patient 3	degree 4 (Normal)	degree 1 (Normal)
Patient 4	degree 3 (Weak)	degree 1 (Weak)

**Table:** (b) Distigmine bromide in patients without BOO (*n* = 3).

	Detrusor contractility (LinPURR)	
	Pre-treatment	Post-treatment
Patient 1	Weak (degree 1)	Normal (degree 1)
Patient 2	Weak (degree 1)	Weak (degree 1)
Patient 3	Weak (degree 1)	Weak (degree 1)

^(1)^BOO, bladder outlet obstruction ^(2)^LinPURR, linear passive urethral resistance relation.

## Abbreviations

BOO: Bladder outlet obstruction
DUA: Detrusor underactivity
IPSS: The International Prostate Symptom Score
PVR: Postvoid residual urine volume
Qmax: Maximum flow rate
LinPURR: Linear passive urethral resistance relation
LUTS: Lower urinary tract symptoms.

## References

[1] Abrams P (1994). In support of pressure flow studies for evaluating men with lower urinary tract symptoms. *Urology*.

[2] Wheeler JS, Culkin DJ, Walter JS, Flanigan RC (1990). Female urinary retention. *Urology*.

[3] Massey JA, Abrams PH (1988). Obstructed voiding in the female. *British Journal of Urology*.

[4] Low BY, Liong ML, Yuen KH (2008). Terazosin therapy for patients with female lower urinary tract symptoms: a randomized, double-blind, placebo controlled trial. *Journal of Urology*.

[5] Tanaka Y, Masumori N, Itoh N, Furuya S, Nishizawa O, Tsukamoto T (2001). Symptomatic and urodynamic improvement by oral distigmine bromide in poor voiders after transurethral resection of the prostate. *Urology*.

[6] Abrams P, Cardozo L, Fall M (2002). The standardisation of terminology of lower urinary tract function: report from the standardisation sub-committee of the international continence society. *Neurourology and Urodynamics*.

[7] Griffiths DJ, van Mastrigt R, Bosch R (1989). Quantification of urethral resistance and bladder function during voiding, with special reference to the effects of prostate size reduction on urethral obstruction due to benign prostatic hyperplasia. *Neurourology and Urodynamics*.

[8] Griffiths DJ, Constantinou CE, van Mastrigt R (1986). Urinary bladder function and its control in healthy females. *American Journal of Physiology*.

[9] van Mastrigt R (1989). Urodynamic analysis using an on-line computer. *Neurourology and Urodynamics*.

[10] Schäfer W (1990). Principles and clinical application of advanced urodynamic analysis of voiding function. *Urologic Clinics of North America*.

[11] Kuo HC (2005). Videourodynamic characteristics and lower urinary tract symptoms of female bladder outlet obstruction. *Urology*.

[12] Diokno AC, Hollander JB, Bennett CJ (1984). Bladder neck obstruction in women: a real entity. *Journal of Urology*.

[13] Axelrod SL, Blaivas JG (1987). Bladder neck obstruction in women. *Journal of Urology*.

[14] Mokhless I, Gaafar S, Fouda K, Shafik M, Assem A (2006). Botulinum a toxin urethral sphincter injection in children with nonueurogenic neurogenic bladder. *Journal of Urology*.

[15] Barendrecht MM, Oelke M, Laguna MP, Michel MC (2007). Is the use of parasympathomimetics for treating an underactive urinary bladder evidence-based?. *BJU International*.

[16] Chassagne S, Bernier PA, Haab F, Roehrborn CG, Reisch JS, Zimmern PE (1998). Proposed cutoff values to define bladder outlet obstruction in women. *Urology*.

[17] Nitti V, Tu LM, Gitlin J (1999). Diagnosing bladder outlet obstruction in women. *Journal of Urology*.

[18] Lemack GE, Zimmern PE (2000). Pressure flow analysis may aid in identifying women with outflow obstruction. *Journal of Urology*.

[19] Blaivas JG, Groutz A (2000). Bladder outlet obstruction nomogram for women with lower urinary tract symptomatology. *Neurourology and Urodynamics*.

